# Dietary supplement guava leaf extract regulates growth, feed utilization, immune function, nutrient digestibility and redox regulation in growing rabbits

**DOI:** 10.1007/s11250-024-04126-4

**Published:** 2024-10-03

**Authors:** Islam G. Abdelghani, Asmaa M. Sheiha, Sameh A. Abdelnour, Mohamed F. Abo El-Maati, Abdelhalim A. El-Darawany, Khaled M. Al-Marakby

**Affiliations:** 1 Department of Animal Production, Faculty of Agriculture, Zagazig University, Zagazig, 44511 Egypt; 2 Agriculture Biochemistry Department, Faculty of Agriculture, Zagazig University, Zagazig, 44511 Egypt

**Keywords:** Growing rabbits, Byproduct phytogenic, Health and nutrient digestibility, Blood physiology

## Abstract

The use of agricultural waste in animal production has gained global interest. An eight-week trial was conducted to investigate the impacts of adding ethanolic guava leaf extract (GLE) as a feed supplement on the growth, feed utilization, immune response, nutrient digestibility, redox regulation, and blood health of growing rabbits. Ninety weaned growing rabbits were randomly assigned to three groups. The first group was fed a basal diet (GLE0), while the other two groups were fed the control diet fortified with 15 mg (GLE15) or 20 mg (GLE20) of GLE per kg of diet for 8 weeks. The HPLC analysis of GLE exhibited the presence of gallic acid, ferulic acid, catechin, and caffeic acid in significant amounts. The results indicated that final body weight, daily body weight, daily feed intake and nutrient digestibility were significantly higher in the GLE-treated groups compared to the un-treated group (*p* < 0.05). Dietary supplementation of GLE significantly reduced lipid contents including triglycerides, total cholesterol, LDL, HDL, and VLDL (*P* < 0.05), with the most significant results observed when adding 20 mg/kg to the diet. AST and ALT levels as well as cortisol hormone in rabbits fed GLE were lower than those in the GLE0 group (*P* < 0.05). Immunoglobulins (IgG and IgA), antioxidant biomarkers (SOD and TAC) and T3 hormone were significantly improved by GLE supplementation (*P* < 0.001). Rabbits fed with GLE had lower levels of ROS and MDA compared to those in the GLE0 group (*P* < 0.001). Moreover, the hepatic and intestinal architectures were maintained in all rabbits fed diets with GLE. The results suggest that GLE supplementation (20 mg/kg diet) in fattening rabbit diets could efficiently improve growth, health status, blood physiology, antioxidant capacity and tissue histology.

## Introduction

Agro-industrial processing worldwide generates tons of byproducts, often leading to pollution due to improper disposal (Freitas et al. 2021). These byproducts contain valuable bioactive compounds that could be used for various purposes, including preventing oxidative stress and promoting general health (Quintero-Herrera et al. 2023; Reguengo et al. 2022). Finding sustainable ways to utilize and treat these byproducts is essential for economic and environmental reasons. In Egypt and several other countries, large amounts of agro-industrial waste are generated, posing environmental risks if not properly managed (Gaur et al. 2020). The animal feed industry has started utilizing these by-products, which can help bridge the nutritional gap in animal diets and reduce feed costs (Quintero-Herrera et al. 2023). Research on alternative feed sources, including agro-industrial wastes, is gaining importance for sustainable livestock production (Mahasneh et al. 2023). Additionally, utilizing these by-products in animal feed not only benefits the economy but also promotes environmental sustainability (Georganas et al. 2023; Nobre et al. 2021). The guava industry's cultivation has led to an environmental issue with the disposal of leaf waste. Environmentalists are concerned about the pollution caused by agro-industries' waste disposal, highlighting the need for proper waste management (Archundia Velarde et al. 2020, Nobre et al. 2021). Guava (*Psidium guajava* Linn) is a prevalent fruit plant found in humid and subtropical zones. Its leaves have been used as a herbal tea in China, India, Egypt, and other countries due to their various bioactivities and health benefits (Sam Arul Raj et al. 2023). Guava leaves contain antioxidants and antibacterial molecules like tannins, saponins, phlorotannins, alkaloids, polyphenols and terpenoids. These compounds, including lycopene, quercetin, and vitamin C, help in fighting metabolic syndromes, respiratory disorders and other diseases (Kumar et al. 2021; Wang et al. 2021b). Guava leaf extract (GLE) is also used in food preservation to prolong the shelf life of fruits. It has powerful antibacterial and antioxidant properties against bacterial strains like *E. coli, Staphylococcus, Shigella,* and *Clostridium* (Carolino et al. 2022; Sam Arul Raj et al. 2023). The biological activities of GLE make it more suitable for promoting the growth and sustaining the health and welfare of growing animals. A meta-analysis conducted by Kumar et al. (2021) provided detailed information about the constituents and biological activities of guava leaves. These activities include antioxidant, antimicrobial, anti-inflammatory, antibacterial, and anti-diarrheal effects (Golovinskaia and Wang 2023; Kumar et al. 2021; Mazumdar et al. 2015), which have been observed in model animals or *in vitro* experiments. The remarkable biological activities of guava leaves make them a potential replacement for antibiotics in animal feeds (Kumar et al. 2024). A study by Nobre et al. (2020) found that guava agro-industrial waste has been incorporated into the diets of finishing lambs up to 30% of the diets. In a study on broiler chickens, Daing et al. (2021) discovered that adding guava leaf meal (1-2% of the diet) enhanced growth, health, and reduced lipid levels. Limited knowledge exists regarding the comprehensive effects of GLE on certain blood biochemical parameters and the growth performance of growing rabbits (Morsy et al. 2019). However, the study did not evaluate immunity, hormonal effects, or histological architecture in New Zealand White rabbits. This research aims to analyze the chemical composition of GLE using GS/Mass. Due to the abundance of phytochemical compounds found in GLE, it exhibits several biological activities such as antioxidant, anti-inflammatory, and immunomodulatory effects. Therefore, this research hypothesized that GLE can enhance blood physiology, nutrient digestibility, organ histology, and immune ability of growing rabbits. Thus, this study investigates the effects of supplementing the diet of fattening rabbits with GLE on their growth, nutrient digestibility, blood physiology, immune response, and intestinal and hepatic architectures.

## Material and methods

### Preparation of ethanolic guava leaf extract and HPLC analysis

The guava leaves were sourced from the local market in Zagazig city. The leaves were gently rinsed with double distilled water, dried in the lab condition, and then powdered using a blender.

The powder was sieved through an aluminum sieve with a 1 mm mesh size to ensure uniform particle size. Approximate, 100 g of the dried leaves powder were kept in sterile Erlenmeyer flask, then 1.5 L of pure ethanol (94.0% purity) was added for 4 days at room temperature. Subsequently, hydroethanolic extracts were prepared using varying water: ethanol ratios (70:30, 50:50, 30:70, and 10:90 v/v). After the 4-day extraction period, the extracts were filtered through Whatman filter paper No. 4 and concentrated using a rotary evaporator at 50 °C. The resulting filtrates were freeze-dried and stored at -18 °C for further investigation. The phenolic compounds in the ethanol extract of guava leaves were assessed using an HPLC approach, as previously described by Saffoon et al. (2014).

### Animals and experimental diets

The present research was conducted at the Rabbit Farm Unit, Zagazig University, Egypt. Ninety male growing New Zealand White rabbits (with an average body weight of 648.59±10.23 grams at 5 weeks of age) were divided into three experimental groups, with 30 rabbits in each group.

The weaned rabbits were housed in standard battery cages (50 × 45 × 40 cm^3^) in a naturally ventilated facility. Each rabbit was housed individually and considered as a replicate.

The composition and nutrient content of the formulated diets can be found in Table 1, meeting the nutritional requirements for growing rabbits as outlined by (De Blas 2010). Water and feeds were available *ad libitum*, and the lighting program provided 16 h light and 8 h dark every day. This experiment was approved by the Institutional Animal Care and Use Committee (IACUC) of Zagazig University under protocol No. ZU-IACUC/2/F/367/2022. The IACUC was followed the guidelines outlined in the Guide for the Care and Use of Laboratory Animals, 2^nd^ Edition, 2022. Efforts have been made to minimize stress and improve animal welfare during the study period.

**Table 1:** Formulation and chemical components of the basal diet (on dry matter basis %)

Ingredients	%
Berseem hay (15%)	30.05
Wheat bran	21.50
Yellow corn	11.00
Soybean meal (44% CP)	17.50
Barley	13.6
DL-Methionine	0.20
Dicalcium phosphate	1.60
Salt	0.30
Molasses	3.00
Limestone	0.95
Mineral and vitamin mixture*	0.30
Proximate chemical composition of the basal diet (on DM basis)	
Items	%
Dry matter	90.62
Organic matter	91.56
Crude protein	19.69
Ether extract	2.56
Crude fiber	13.33
Nitrogen free extract	55.97
Ash	8.44
DE (Kcal/kg DM) **	2555

*: minerals and vitamins mixture. Each kilogram diet contained: vitamin A, 6000 IU, vitamin D3, 9000 IU, vitamin E, 40 mg, vitamin K3, 2 mg; vitamin B1, 2 mg; vitamin B2, 4 mg, , pantothenic acid, 10 mg, vitamin B6, 2 mg vitamin B12, 0.01 mg; biotin, 0.05 mg, folic acid, 3 mg, niacin, 50 mg, choline, 250 mg, Fe, 50 mg, Zn, Cu, 5 mg, 50 mg, Mn, 85 mg, Se, 0,1 mg, Co, 0.1 mg, and I, 0.2 mg.

**: Digestible energy, evaluated corresponding to (Fekete and Gippert 1986) method as follows:

DE (Kcal/Kg DM) = 4253-32.6 (CF%) -144.4 (Total ash)

### Experimental design and sampling

The growing rabbits were divided into three experimental groups. Group 1 (GLE0) consisted of rabbits fed a commercial diet *ad libitum* and served as the control group. Group 2 (GLE15) included rabbits fed a commercial diet supplemented with 15 mg/kg of GLF. Group 3 (GLE20) comprised rabbits fed a commercial diet supplemented with 20 mg/kg of GLF, following the recommendations from Shakal et al. (2024). The trial lasted for 8 weeks (6-13 weeks of age). At the end of this period, six rabbits from each group were randomly selected for blood sampling, tissue examination, and carcass analysis.

The rabbits were fasted overnight, before blood sampling. Blood samples were collected and transferred into two tubes. The first tube contained EDTA (anticoagulant) for assessing blood hematology. The other one without EDTA for serum separation and kept at room temperature for 1 hour to separate the serum. Then the samples were centrifuged at 4000 rpm for 15 minutes. The collected serum samples were stored in a deep freezer at approximately -20 ºC until biochemical analysis. The fasted rabbits (n=6) were randomly selected from each group and slaughtered closely after weighing corresponding to the Islamic procedures to extract the internal tissues for histological examination and carcass traits assessment.

### Evaluating growth indices and carcass traits

The body weight (BW) of all tested rabbits was recorded at the start of the test and examined weekly. The quantity of feed presented and refused was recorded and balanced every day throughout the entire investigation period. The body weight gain (BWG), daily feed intake (DFI), and feed conversion ratio (FCR) were evaluated. The method described in detail by Abdelnour et al. (2023) was used to determine the growth indices as follows: BWG (g) = final weight (g) – initial weight (g); ADG (g/d) = BWG (g)/experimental day (d); FCR = DFI (g/d)/average daily gain (g/d). The slaughtering and carcass analysis processes were conducted in accordance with the Islamic Method. Non-edible parts such as pelt, tail, and viscera were removed after bleeding. The carcass and edible parts (kidney, heart, and liver) along with the spleen, head, and lung were weighed (g). The dressing percentage was calculated as the ratio of the hot-dressed carcass weight to pre-slaughter weight, stated as a percentage. The cecum length (cm) was also measured.

### Measuring blood hematology and serum metabolites

The blood samples were assessed using an automated hematology analyzer (Hospitex Hema Screen 18, Sesto Fiorentino, Italy) following the method described by Moore et al. (2015).

Blood serum metabolites, including total protein (TP), low-density lipoprotein (LDL), alanine transaminase (ALT), uric acid, triglycerides, aspartate aminotransferase (AST), creatinine, albumin, total and direct bilirubin, total cholesterol, and high-density lipoprotein (HDL) were assessed spectrophotometrically using commercial kits offered by Bio-diagnostic Company (Giza, Egypt), following the manufacturer's directions. The VLDL (very low-density lipoprotein) levels were determined based to the previous method of Okada et al. (1998), using the levels of total triglycerides. The difference between the TP and albumin was used for calculating the serum globulin levels. Glucose levels in the rabbit serum were assessed using an enzymatic hexokinase oxidase reaction on the mentioned analyzer (Selleri et al. 2014). All biochemical analyses were detected using the Dimension® EXL™ 200 Integrated Chemistry System.

### Redox status, immune response, and hormone profile

To assess antioxidant profile and oxidative stress parameters, the activities of superoxide dismutase (SOD) (Marklund et al. 1982) and total antioxidant capacity (TAC) (Ghiselli et al. 2000), as well as the levels of malondialdehyde (MDA) (Alipour et al. 2006) and reactive oxygen species (ROS) (Dandona et al. 2001) were measured in rabbit serum using commercial kits and a spectrophotometer (Shimadzu, Kyoto, Japan). Blood serum levels of T3 (triiodothyronine) were determined according to Abdel-Fattah et al. (2011), by radioimmunoassay (RIA). The serum levels of immunoglobulins (IgG and IgA) were determined following the method defined by (Bergmann-Leitner et al. 2008). Serum cortisol levels were determined by RIA using commercial CORT kit (ICN Biomedical Inc., USA) following the standard procedures described by the manufacturing company (Abdelnour et al. 2023).

### Digestibility coefficients and feeding values

The digestibility study spanned four days (10 animals from each group). During this period, final FI was recorded, and the total feces from the 10 replicates were collected, weighed, and stored in a freezer at -10 °C until preparation for chemical analysis. The fecal samples were partially dried at 60 °C for 48 hours before analysis. Apparent nutrient digestibility was calculated using the classical formula, where NI represents nutrient intake and NE represents fecal nutrient excretion, resulting in apparent nutrient digestibility coefficients.

### Chemical analysis

The diets, well-dried fecal samples were ground to pass through a 1 mm screen using a grinder. Subsequently, samples of the feed and feces were examined for moisture content by oven drying (method No. 930.15), protein content by Kjeldahl (method No. 984.13), ash by incineration (method No. 942.05), crude fiber (method No. 978.10), and ether extract by Soxhlet fat analysis (method No. 954.02), as performed by the AOAC International guidelines (AOAC 2000). Nitrogen-free extract (NFE) was estimated by subtraction. Digestible energy (DE) was estimated according to Fekete and Gippert (1986), utilizing the subsequent equation: DE (Kcal/Kg DM) = 4253-32.6 (CF%) -144.4 (Total ash).

### Histology examination

Intestinal and hepatic specimens from animals in the GLE0, GLE15 and GLE20 groups were collected for histological assessment. These specimens were fixed in a neutral formalin buffer (10%). Subsequently, the samples underwent dehydration in increasing concentrations of ethyl alcohol, followed by clearing in two changes of xylene. They were then embedded in paraffin blocks and sectioned at 4-μm thickness using a microtome (Leica RM 2155). All sections were stained with eosin and hematoxylin following the method described by Bancroft and Gamble (2008). A digital camera microscope (Leica DM 500, Leica EC3, Leica) was utilized to capture representative images.

### Statistical analysis

The data were analyzed using the general linear model, specifically the one-way analysis of variance (ANOVA), with the diet serving as the fixed factor. The data was analyzed using the SPSS (version 25, SPSS Inc., Chicago, IL, USA). The following mathematical model was used: Yij = μ+ TRTi + eij. Where Yij = Observations, μ = Overall mean, TRT = effect of the GLE (i, 15 or 20 mg/kg diet), eij = random error. Results were expressed as mean ±SE. Duncan's test was used to perform multiple comparisons between means in case a significant effect was detected. The statistical significance was considered at *P*<0.05.

## Results

### Detection of phenolic molecules

**Fig. 1:**
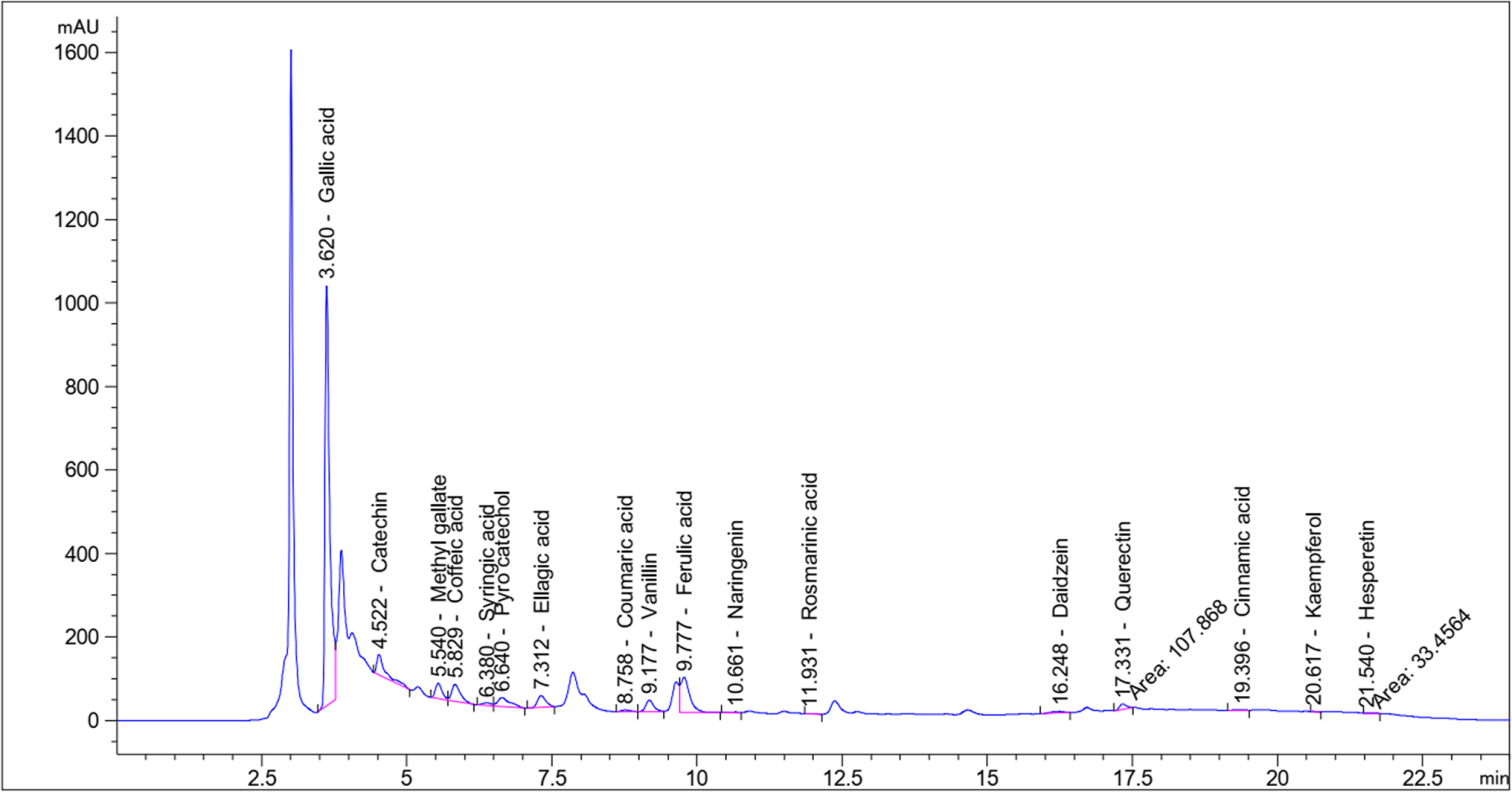
The HPLC analysis of GLE. The most abundant phenolic compounds such as gallic acid, ferulic acid, catechin, and caffeic acid were identified

The GLE was analyzed by HPLC, and 18 phenolic compounds were identified (Table 2 and Fig. 1). The HPLC exploration of GLE exhibited the existence of gallic acid, ferulic acid, catechin, caffeic acid in high quantities (Fig. 1, Table 2). Additionally, the analysis of phenolic content revealed that the GLE contains a substantial number of phenolic compounds, which are directly associated with its capability to scavenge free radicals in various cellular systems.

**Table 2:**
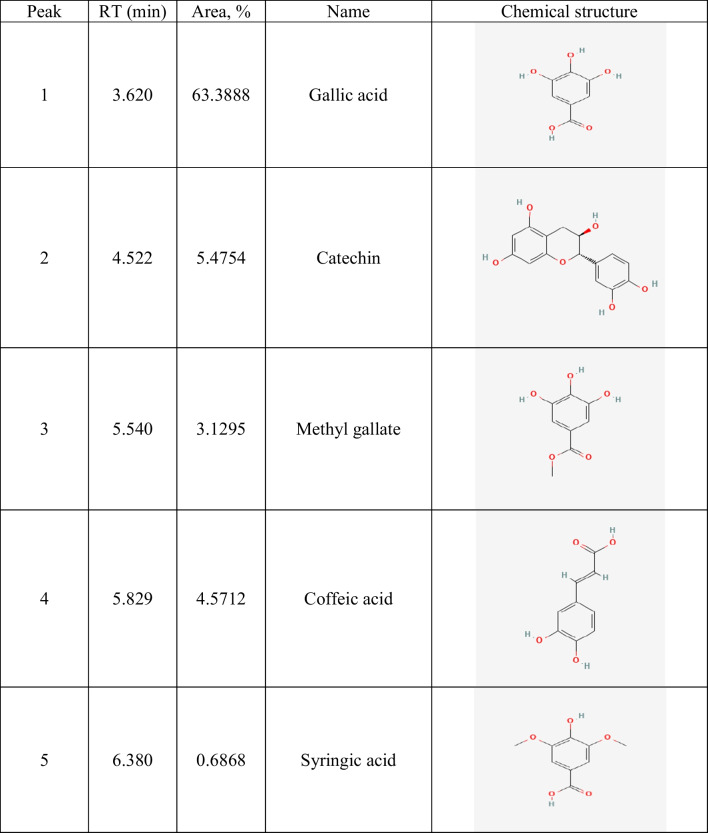

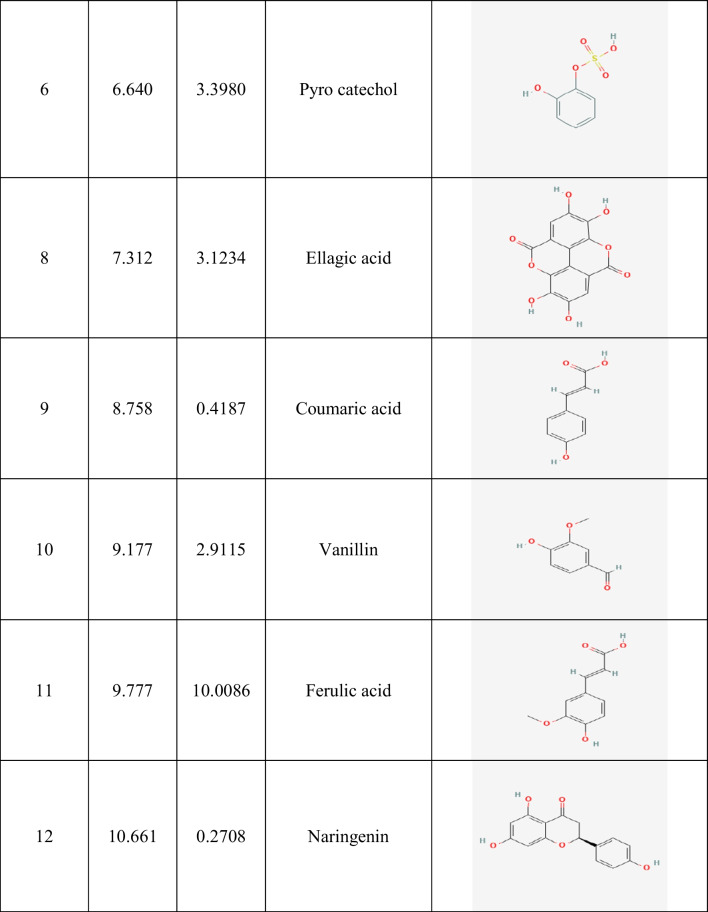

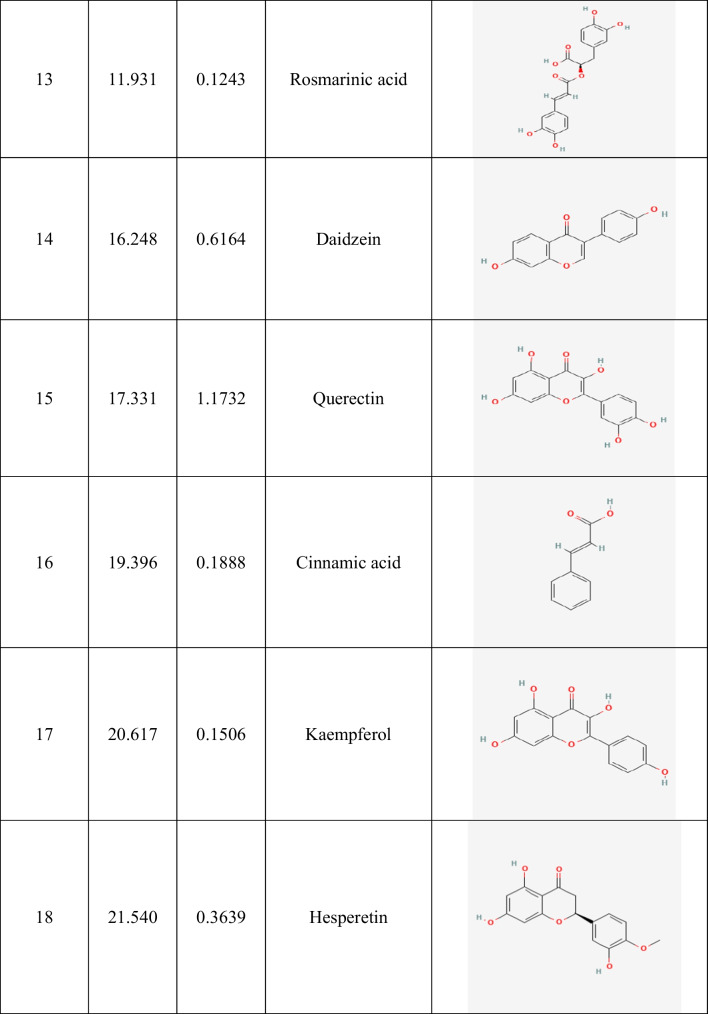
Identification of phenolic compounds in ethanolic guava leaf extract (GLE) by HPLC

### Growth performance and carcass indices

The heaviest final body weight was observed in the GLE20 group, followed by the GLE15 group compared to the free-GLE diet (P<0.05; Table 3). Rabbits in the GLE20 and GLE15 groups had 12.51% and 9.07% greater body weight (BW), respectively, compared to the free-GLE diet. All GLE groups showed better daily weight gain (DWG) than the control group (P<0.05). Feed intake (FI) was significantly improved by dietary GLE supplementation (P<0.05), while feed conversion ratio (FCR) was not affected by the treatment (P=0.427). The highest values of hot carcass, liver, spleen, and heart weights were detected in the GLE20 group (P<0.05), while GLE15 exhibited intermediate values compared to other groups (P>0.05; Table 3). The weights of lung, fur, kidneys, cecum weight and length, and gastrointestinal tract of growing rabbits were non-significantly influenced by the dietary GLE supplementation (P>0.05).

**Table 3:** Growth performance and carcass indices of growing male rabbits fed diets containing ethanolic guava leaf extract (GLE, 0, 15 and 20 mg/kg diet)

Items	Treatments^1^			Pooled SEM	*P*-value
	GLE0	GLE15	GLE20		
Initial body weight, g	638.46 ± 13.16	658.85 ± 12.61	648.46 ±11.26	7.080	0.513
Final body weight, FBW, g	1946.15^**c**^ ± 9.48	2122.69^**b**^±19.69	2189.62^**a**^±21.21	19.36	< 0.001
Daily weight gain, BWG,	18.68^**b**^ ± 0.18	21.13^**a**^ ± 0.29	21.68^**a**^ ± 0.43	0.277	< 0.001
Daily feed intake, DFI g	82.37^**c**^± 1.63	89.29^**b**^ ± 0.91	92.32^**a**^ ± 1.23	0.991	< 0.001
Feed conversion ratio, %	4.42 ± 0.10	4.23 ± 0.07	4.28 ± 0.12	0.057	0.427
Hot carcass, g	1120.00^**b**^±52.20	1201.67^**ab**^±51.9	1325.00^**a**^±22.55	37.18	0.046
Liver, g	53.67^**b**^±1.97	69.05^**a**^±3.95	73.19^**a**^±0.83	3.24	0.004
Lung, g	13.69±0.93	13.58±1.08	14.74±0.54	0.48	0.614
Spleen, g	0.67^**b**^±0.04	0.74^**ab**^±0.03	0.79^**a**^±0.01	0.02	0.074
Kidneys, g	13.19±0.96	13.13±0.87	15.39±0.37	0.54	0.145
Heart, g	5.35^b^±0.66	6.68^**ab**^±0.30	7.72^**a**^±0.41	0.42	0.036
Fur, g	340.00±5.77	346.67±1.67	346.67±9.28	3.38	0.709
Gastrointestinal tract, g	251.67±31.80	273.33±16.91	278.33±13.33	11.82	0.682
Cecum weight, g	5.70±0.76	9.73±1.15	8.66±1.80	0.89	0.159
Cecum length, cm	9.83±0.60	11.50±0.50	12.00±1.15	0.52	0.218

^1^GLE0 group: rabbits fed basal diet without additives. GLE15 and GLE20 groups: rabbits fed basal diet supplemented with 15 mg and 20 mg/kg diet, respectively. ^a,^ ^b^ ^and^ ^c^ Means with different superscript in the same row are significantly different (*P* ≤0.05)

### Digestibility coefficients and feeding values

There was no statistical difference among groups in organic matter, ether extract, and crude fiber (*P* > 0.05; Table 4). GLE20 significantly increased the dry matter compared to other treated groups (*p* < 0.05). All GLE improved the percentages of nitrogen-free extract and crude protein with non-significant differences among GLE0 and GLE15 groups (*P* > 0.05). DE values were not affected by the GLE dietary inclusion (*P* > 0.05). TDN and DCP of growing rabbits fed 20 mg of GLE were greater than those of the free-GLE group (*P* < 0.05), while GLE15 exhibited a non-significant effect on TDN and CB compared to the control group (*P* > 0.05).

**Table 4:** Effects of dietary inclusion of GLE on the digestibility coefficients and feeding values of rabbits

Items	Treatments^1^			Pooled SEM	*P*-value
	GLE0	GLE15	GLE20		
Digestibility coefficients %					
Dry matter	65.17^b^ ± 1.93	67.35^b^ ± 2.38	73.75^a^ ± 0.98	1.34	0.014
Organic matter	69.73 ± 3.47	72.44 ± 1.97	75.40 ± 2.78	1.62	0.385
Crude protein	69.72^b^ ± 2.18	72.45^ab^ ± 2.38	76.07^a^ ± .61	1.21	0.091
Ether extract	72.73 ± 2.25	77.05 ± 1.67	78.26 ± 1.47	1.15	0.113
Nitrogen free extract	72.22^b^ ± 2.22	75.02^ab^ ± 1.48	78.91^a^ ± 1.25	1.14	0.043
Crude fiber	65.67 ± 2.75	68.97 ± 1.14	70.67 ± 1.55	1.17	0.213
Feeding values					
TDN*	72.77^b^ ± 2.31	74.05^b^ ± 1.81	80.12^a^ ± 1.12	1.25	0.026
DCB**	13.75^b^ ± 0.44	14.27^ab^ ± 0.47	14.94^a^ ± 0.10	36.77	0.109
DE *** Kcal/kg DM	2236.08±33.88	2359.21±73.25	2382.66±69.29	0.24	0.226

^1^GLE0 group: rabbits fed basal diet without additives. GLE15 and GLE20 groups: rabbits fed basal diet supplemented with 15 mg and 20 mg/kg diet, respectively. TDN*: Total Digestible Nutrients TDN = % DCP + % DCF + % DNFE + (2.25 x % DEE) , DCP**: Digestible crude protein DCP = Digestibility Co-efficient × CP content of the feed DE***: Digestible energy Calculated according to Fekete and Gippert (1986) as follows : DE (Kcal/Kg DM) = 4253- 32.6 (CF%)- 144.4 (Total ash). ^a^ ^and^ ^b^ Means with different superscript in the same row are significantly different (*P* ≤0.05)

### Blood hematology

The impacts of GLE on blood hematology in growing rabbits are clarified in Table 5. The PCV and MCV values were higher in the GLE20 group compared to the GLE15 and GLE0 groups (*P* < 0.05). Other erythrogram indices were not affected by dietary GLE supplementation (*P* > 0.05). Neutrophils were significantly reduced, while lymphocytes were significantly increased with the addition of GLE to the rabbit diets (*P* < 0.05). The percentages of basophils and monocytes were higher in the GLE15 group compared to the other groups (*P* < 0.05). GLE20 significantly decreased the levels of eosinophils compared to the other groups (*P* < 0.05).

**Table 5:** The changes of haematological profile of growing rabbits fed diets containing ethanolic guava leaf extract (GLE, 0, 15 and 20 mg/kg diet)

Items^2^	Treatments^1^				Pooled SEM	*P*-value
	GLE0	GLE15	GLE20			
HCT, %	33.87±1.69	34.84±1.12	37.45±0.72		0.82	0.190
PCV, %	33.31^**b**^±1.16	35.42^**b**^±1.37	39.95^**a**^±1.18		1.16	0.023
RBCs, ×10^6^/μl	4.58±0.37	4.78±0.127	4.92±0.16		0.08	0.210
Hb, g/dl	11.54±0.53	11.84±0.29	12.86±0.68		0.33	0.252
MCV, fL	72.76^**b**^±2.06	74.16^**b**^±2.29	81.20^**a**^±1.50		1.64	0.049
MCHC, µm^3^	34.67±1.53	33.47±0.46	32.19±1.32		0.70	0.401
MCH, pg	25.19±0.98	24.80±0.54	26.10±0.62		0.42	0.485
PLT, ×10^3^/μl	187.33±6.39	190.33±6.06	193.33±4.33		2.96	0.765
WBCs, ×10^3^/μl	8.29±0.68	8.15±0.37	8.28±0.59		0.28	0.982
WBCs fractionation %						
NEU, %	41.37^**a**^ ± 0.60	35.24^**b**^±0.35		23.15^**c**^±0.592	2.69	**<** 0.001
LYM, %	54.67^**c**^ ± 0.58	60.19^**b**^±0.32		72.90^**a**^±0.668	2.71	**<** 0.001
Eso, %	0.26^**a**^ ± 0.01	0.26^**a**^±0.01		0.22^**b**^±0.009	0.01	0.017
MON, %	3.41^**b**^ ± 0.06	3.94^**a**^±0.09		3.42^**b**^±0.145	0.10	0.018
BAS, %	0.29^**b**^ ± 0.01	0.36^**a**^±0.01		0.31^**b**^±0.015	0.01	0.015

^1^GLE0 group: rabbits fed basal diet without additives. GLE15 and GLE20 groups: rabbits fed basal diet supplemented with 15 mg and 20 mg/kg diet, respectively. ^2^ HCT: Hematocrit, MCHC: Mean corpuscular hemoglobin concentration, RBC: Red blood cells, Hb: Hemoglobin, WBC: White blood cells, PCV: packed cell volume, LYM: Lymphocytes, MCV: Mean corpuscular volume, PLT: platelets, NEU: Neutrophils, MCH: Mean corpuscular hemoglobin, Eso: Eosinophils, MON: Monocytes, BAS: Basophils. ^a, b and c^ Means with different superscript in the same row are significantly different (*P* ≤0.05)

### Bood metabolites

Dietary GLE supplementation significantly reduced lipid contents such as triglycerides, total cholesterol, HDL, and LDL (*P* < 0.05; Table 6), with the best results seen when adding 20 mg/kg to the diet (Table 6). Creatine and uric acid levels were not different among the treated groups (*P* > 0.05). Similarly, total bilirubin, total protein, direct bilirubin, A/G ratio, albumin, indirect bilirubin, globulin, and glucose values were not significantly affected by dietary GLE supplementation (*P* > 0.05). AST and ALT levels in rabbits fed GLE were lesser than those in the GLE0 group (*P* < 0.05). VLDL levels were significantly improved by dietary GLE addition (*P* < 0.05).

**Table 6:** Blood metabolites variables of growing rabbits fed diets containing ethanolic guava leaf extract (GLE)

Items^2^	Treatments^1^			Pooled SEM	*P*-value
	GLE0	GLE15	GLE20		
Total Protein, g/dL	5.07 ± 0.04	5.05 ± 0.07	5.08 ± 0.10	0.04	0.962
Albumin, g/dL	2.65 ± 0.04	2.67 ±0.07	2.70 ± 0.10	0.02	0.736
Globulin, g/dL	2.42 ± 0.04	2.38 ± 0.06	2.38 ± 0.04	0.03	0.915
A/G, %	1.10 ± 0.03	1.12 ± 0.03	1.14 ± 0.05	0.02	0.756
Glucose, mg/dL	76.34 ± 30.06	105.97 ± 1.67	106.61 ± 1.35	10.03	0.426
Triglycerides, mg/dL	90.51^**a**^ ± 2.12	73.88^**b**^ ± 1.36	68.22^**b**^ ± 1.57	3.45	< 0.001
Total cholesterol, mg/dL	93.49^**a**^ ± 0.99	82.79^**b**^ ± 6.69	70.67^**c**^ ± 2.87	3.48	< 0.001
HDL, mg/dL	39.79^**a**^ ± 1.30	36.55^**b**^ ± 1.59	36.11^**b**^ ±1.81	0.74	0.059
LDL, mg/dL	36.39^**a**^ ± 0.53	30.53^**b**^ ± 1.16	20.91^**c**^ ± 1.99	2.38	< 0.001
VLDL, mg/dL	13.64^**c**^ ± 0.31	15.37^b^ ± 0.20	17.97^**a**^ ± 0.53	0.66	< 0.001
Creatinine, mg/dL	1.14± 0.03	1.13 ± 0.02	1.15 ± 0.03	0.01	0.901
Uric acid, mg/dL	2.92± 0.06	2.84± 0.06	2.89 ± 0.03	0.03	0.588
ALT, U/L	23.65^**a**^ ± 0.71	20.99^**b**^ ± 0.19	20.80^**b**^ ± 0.36	0.52	0.009
AST, U/L	32.39^**a**^ ± 0.98	25.38^**b**^ ± 0.56	25.88^**b**^ ± 0.89	1.20	0.002
Total Bilirubin, mg/dL	0.47 ± 0.03	0.51 ± 0.01	0.50 ± 0.02	0.01	0.320
Indirect bilirubin, mg/dL	0.32 ± 0.03	0.36 ± 0.01	0.35 ± 0.02	0.01	0.334
Direct bilirubin, mg/dL	0.15 ± 0.01	0.14 ± 0.01	0.15 ± 0.01	0.004	0.746

^1^GLE0 group: rabbits fed basal diet without additives. GLE15 and GLE20 groups: rabbits fed basal diet supplemented with 15 mg and 20 mg/kg diet, respectively. ^2^AG: albumin/globulin, HDL: High-density lipoprotein, LDL: Low-density lipoprotein, AST: aspartate aminotransferase, VLDL: Very low-density lipoprotein, ALT: alanine aminotransferase. ^a,^ ^b^ ^and^ ^c^ Means with different superscript in the same row are significantly different (*P* ≤0.05)

### Immune response, redox status, and hormone profile

Immunoglobulins (IgG and IgA) showed significant improvement with GLE supplementation, with the respectable results detected in the GLE20 group (*P* < 0.001, Table 7). The improvement of IgA was 63.84% and 26.41% for the GLE20 and GLE15 groups, respectively, compared to the control group. Antioxidant-related biomarkers SOD and TAC were significantly increased in all GLE-treated groups compared to the GLE0 group (*P* < 0.001). Rabbits fed GLE had lower levels of ROS and MDA compared to those in GLE0 group (*P* < 0.001). Feeding rabbits with GLE at 20 mg/kg diet significantly increased T3 levels compared to other treated groups (*P* < 0.05), while the addition of GLE (15 or 20 mg/kg diet) significantly reduced cortisol hormone levels (*P* < 0.001).

**Table 7:** The changes of immune response, redox status, and hormone profile of growing rabbits fed diets containing ethanolic guava leaf extract (GLE, 0, 15 and 20 mg/kg diet)

Items^2^	Treatments^1^			Pooled SEM	*P*-value
	GLE0	GLE15	GLE20		
Immune response					
IgA, ng/mL	183.89^**c**^ ± 3.78	232.41^**b**^ ± 6.47	301.21^**a**^ ± 12.90	17.55	< 0.001
IgG, ng/mL	279.00^**c**^ ± 0.67	321.21^**b**^ ± 6.66	394.06^**a**^ ± 5.07	16.98	< 0.001
Redox status					
SOD, U/mL	77.25^**b**^ ± 1.92	114.52^**a**^ ± 4.43	123.83^**a**^ ± 6.21	7.47	< 0.001
TAC, ng/mL	1.07^**b**^ ± 0.07	2.57^**a**^ ± 0.08	2.75^**a**^ ± 0.05	0.27	< 0.001
ROS, pg/mL	218.05^**a**^ ± 4.51	182.07^b^ ± 3.67	167.92^**b**^ ± 5.28	7.80	< 0.001
MDA, nmoL/mL	1.32^**a**^ ± 0.06	0.97^**b**^ ± 0.05	0.91^**b**^ ± 0.07	0.07	0.006
Hormone profile					
T3_,_ ng/mL	1.78^**b**^ ± 0.06	1.90^**b**^ ± 0.04	2.24^**a**^ ± 0.04	0.07	< 0.001
Cortisol, ng/mL	44.66^**a**^ ± 1.25	35.56^**b**^ ± 0.74	32.48^**b**^ ± 0.63	1.89	< 0.001

^1^GLE0 group: rabbits fed basal diet without additives. GLE15 and GLE20 groups: rabbits fed basal diet supplemented with 15 mg and 20 mg/kg diet, respectively. T3: Triiodothyronine, IgA: immunoglobulin A, immunoglobulin G: IgG, SOD: Superoxide dismutase, ROS: reactive oxygen species, MDA: malondialdehyde, TAC: total antioxidant capacity. ^a,^ ^b^ ^and^ ^c^ Means with different superscript in the same row are significantly different (*P* ≤0.05)

### Histology examination

**Fig. 2:**
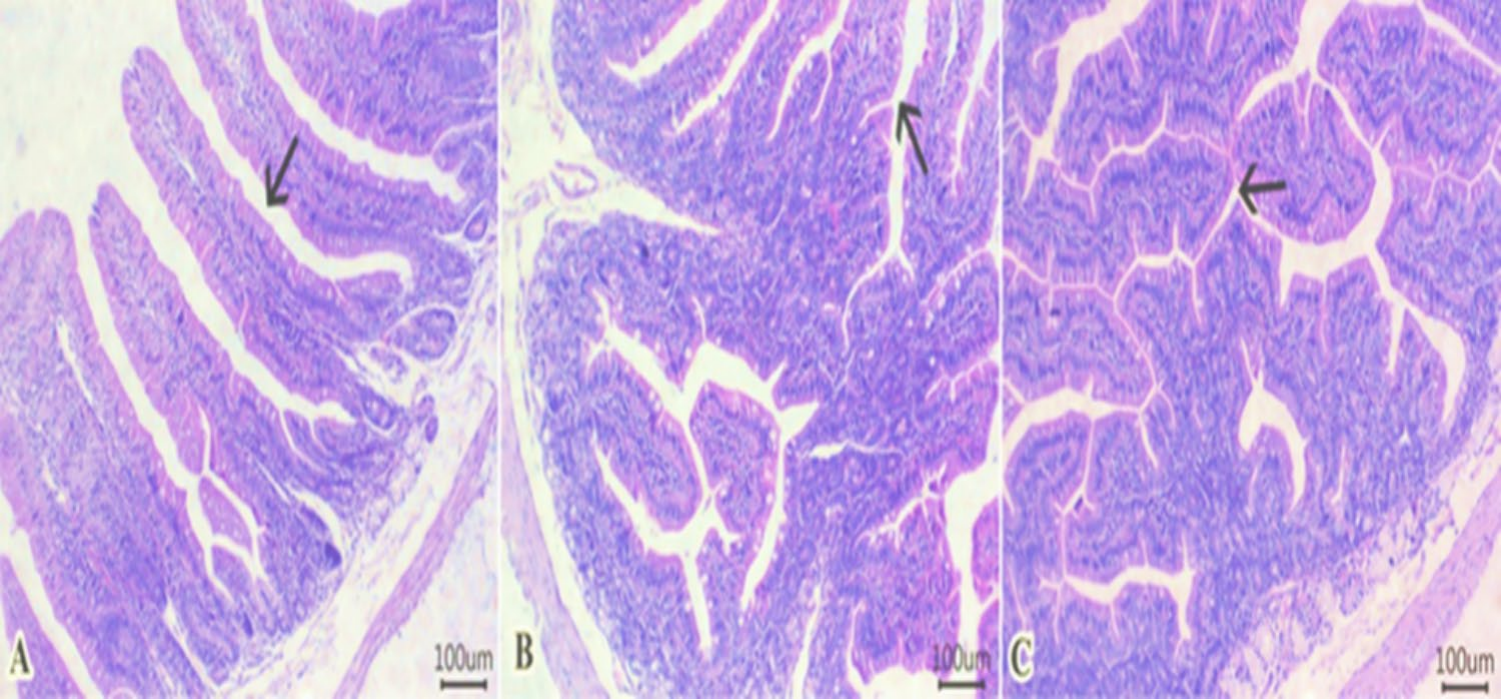
(**A-C**)**.** Photomicrograph of H&E-stained sections from the small intestine of a growing rabbit (duodenum section, Scale bar 100 μm). The rabbit fed with the control diet (Fig. 2A) shows normal histological architecture of the columnar epithelial lining villi (arrow), lamina propria, submucosal layer with prominent submucosal Brunner's glands, muscular layer, and serosa. The treated groups: growing rabbits fed diets with 15 (Fig. 2B) and 20 (Fig. 2C) mg/kg diet exhibited an increase in villous heights with a moderate number of branching villi (arrow) as well as more elongated and branched villous epithelium than rabbits fed the control diet (Fig. 2A)

**Fig. 3:**
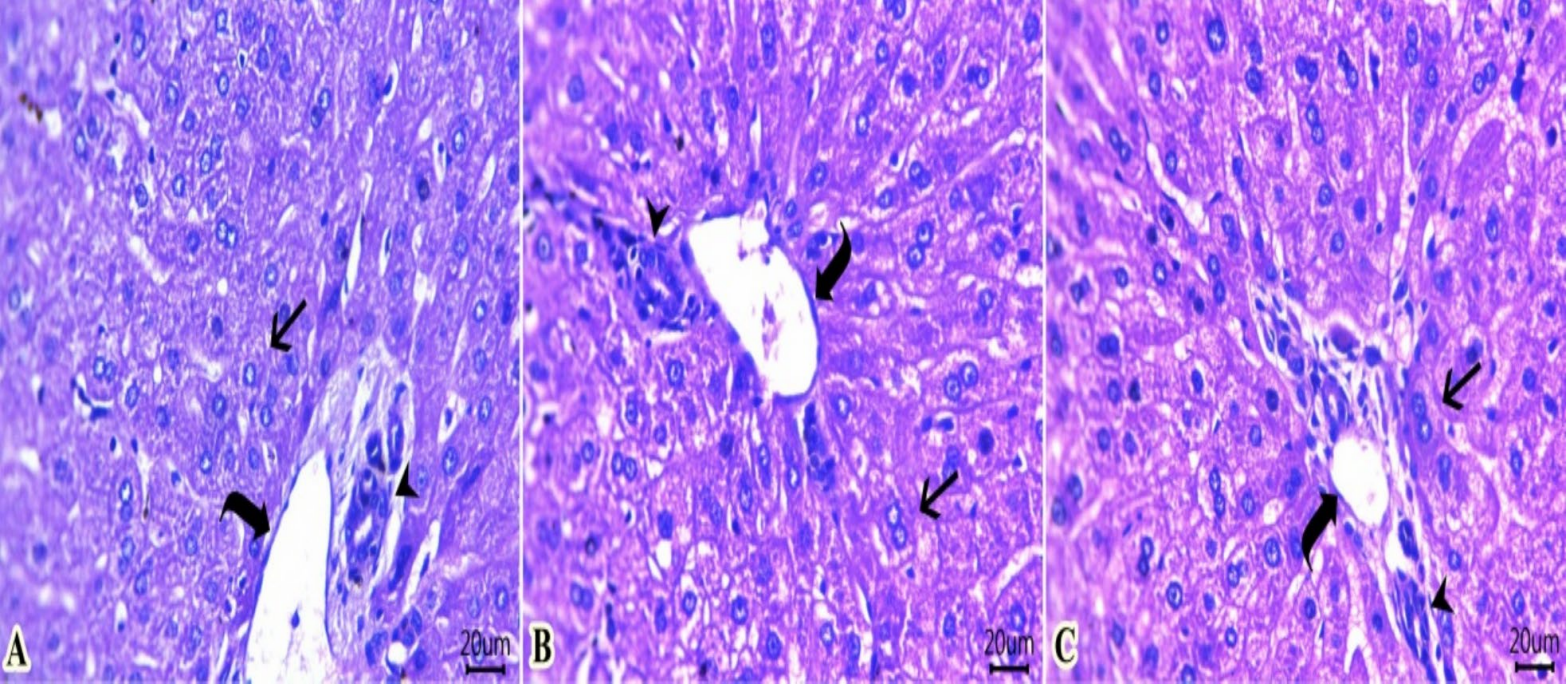
(**A**-**C**). Photomicrograph of H&E-stained sections from the liver of growing rabbits (Scale bar 20 μm). The rabbit in control group (Fig. 3A) exhibited normal histomorphology of hepatic cords (arrow), portal vein (curved arrow), and bile ducts (arrowhead). The liver of growing rabbits showed an increased number of binucleated cells (arrow) along with normal portal vein (curved arrow) and normal bile duct epithelium (arrowhead) in rabbit fed 15 mg of GLE in their diets (Fig. 3B). The intestines of growing rabbits fed 20 mg of GLE in their diets had a greater number of binucleated cells (arrow) alongside preserved configurations of the portal vein (curved arrow) and bile duct (arrowhead) (Fig. 3C)

Rabbits fed a control diet (Fig. 2A) showed normal histological architecture of columnar epithelial lining villi (arrow), lamina propria, submucosal layer with prominent submucosal Brunner's glands, muscular layer, and serosa. The treated groups, growing rabbits fed diets with 15 (Fig. 2B) or 20 (Fig. 2C) mg /kg diet, exhibited an increase in villous heights with a moderate number of branching villi (arrow), as well as more elongated and branched villous epithelium of the intestinal tissues than rabbits fed the control diet (Fig. 2A). The rabbit of control group (Fig. 3A) had normal histomorphology of hepatic cords (arrow), portal vein (curved arrow), and bile ducts (arrowhead). The liver of growing rabbits fed 15 mg of GLE /kg diet had an increased number of binucleated cells (arrow) and normal portal vein (curved arrow) and normal bile duct epithelium (arrowhead) in the group (Fig. 3B). The liver of growing rabbits fed 20 mg of GLE /kg diet had a greater number of binucleated cells (arrow) beside preserved configurations of the portal vein (curved arrow) and bile duct (arrowhead) (Fig. 3C).

## Discussion

Utilizing bioactive compounds from agro-industrial waste to enhance growth and improve animal health, while reducing environmental impact is a sustainable strategy in the livestock industry. Egypt is a main producers of guava in the world, highlighting the importance of adopting sustainable and environmentally friendly practices for guava byproducts (Zahid et al. 2019). However, there is limited research on the use of GLE in the rabbit industry. A study by (Morsy et al. (2019) explored the potential effects of GLE on some blood biochemical parameters and growth performance in APRI rabbits. However, the study did not investigate immunity, hormonal effects, or organ architectures. This paper appears to be the first to comprehensively explore the potential benefits of using GLE to improve the health, growth, and welfare of rabbits during the fattening period.

The results showed that supplementation with GLE (15 or 20 mg/kg diet) significantly boosted the growth performance (DFI, FBW, and BWG), carcass traits (hot carcass, liver, spleen weights), and improved the hepatic and intestinal architectures of growing rabbits. Moreover, GLE administration considerably led to a notable reduction in the lipid profile of growing rabbits due to its anti-hyperglycemic action. Furthermore, GLE exhibited a hepatoprotective effect hepatoprotective effect, as liver enzymes were significantly decreased by feeding GLE (15 or 20 mg/kg diet). Feeding growing rabbits with 20 or 15 mg of GLE resulted in a significant increase in immune response, T3 hormone levels, and antioxidant capacity, while also decreasing levels of MDA, ROS, and cortisol hormone compared to the control group. Rabbits fed diets with GLE for 8 weeks showed higher FBW and body weight gain compared to control diets. However, the GLE supplementation did not affect the FCR (*P* > 0.05). Feed intake was significantly improved with higher levels of GLE in the diets (*P* < 0.001). This improvement in growth performance could be attributed to the antioxidant capacity of GLE. In a study conducted by Ali (2008), it was found that feeding growing rabbits with guava by-product meal (up to 30%) significantly improved growth indices such as FBW and BWG compared to the control group. In a study on broiler chickens, Daing et al. (2021) discovered that adding guava leaf meal (1-2% of the diet) enhanced growth performance. A diet containing 20% guava waste mixed with 1% organic acid or mannan oligosaccharide significantly improved FBW and BWG of growing rabbits (Kamel et al. 2016). Moreover, rabbits fed diets supplemented with 1-2 ml of guava leaf extract per kg of diet showed significant improvements in FBW and BWG (Morsy et al. 2019). The improved growth indices in growing rabbits may be due to the increased digestibility of nutrients and the antimicrobial properties of GLE (Shakal et al. 2024).

Recent searches have highlighted the potential of GLE in treating diarrhea, attracting attention from the scientific community (Dewi et al. 2013; Kumar et al. 2021; Swelum et al. 2021). Reducing diarrhea in growing animals is crucial for maintaining the health, growth, and survival rates in rabbit farms. Other works suggest that the development in growth could be accredited to the antidiarrheal properties of GLE, as confirmed by several authors (Dewi et al. 2013; Mazumdar et al. 2015; Ojewole et al. 2008). Additionally, GLE has antimicrobial properties that can help eliminate harmful gut microbes that may cause diarrhea (Birdi et al. 2020). This can be accredited to the bioactive molecules present in GLE, such as gallic acid, p-coumaric acid, kaempferol, and quercetin, which have been shown to enhance the growth of various animal’s groups. These bioactive constituents enhance FI and rabbit growth by stimulating the excretion of digestive enzymes (North et al. 2018). Gallic acid can promote blood circulation, thereby improving nutrient delivery for cellular uptake (Jin et al. 2018). Further research is needed to explore the molecular mechanisms and long-term safety of GLE for potential therapeutic use in humans.

Using alternative feed ingredients can lower production costs and increase profitability. Guava waste can be incorporated into the diet of fatting rabbits at concentrations of up to 20% without any negative impact on productivity or economic efficiency (Ali 2008, Kamel et al. 2016). A study by Kamel et al. (2016) informed that 20% of guava waste with 1% organic acids substantially improved the apparent digestibility of OM, CP, EE, NDF, and ADF in growing rabbits. In this study, a remarkable increase in the percentages of DM, CP, NFE, and feeding values (TDN and DCP) were recorded in rabbit fed diets containing 20 mg of GLE compared to other groups. The anti-diarrheal properties of GE can be primarily attributed to its flavonoids and tannins. These compounds have the ability to alter the structure of proteins and create interactions with tannates, ultimately leading to a decrease in intestinal mucosal permeability (Wang et al. 2021a; Wang et al. 2021b; Ismail et al. 2019). Furthermore, the flavonoids and phenolics present in GE have strong antibacterial effects against intestinal bacteria such as *Salmonella* and *Escherichia coli* (Sen et al. 2015). This antibacterial activity can help in reducing diarrhea in piglets infected with enterotoxigenic *E. coli* (Wang et al. 2021a, Wang et al. 2021b). Guava leaves are also a rich source of dietary fiber, which can enhance dietary availability and production by modulating the microbiota.

Carcass traits are the end result of an animal's growth period, reflecting the impact of nutritional interventions during fattening. In a study, rabbits fed diets with 20 mg showed better hot carcass weights and liver quality compared to other groups. Liver and heart weights were significantly enhanced in growing rabbits fed diets containing 30% guava by-product meal, consistent with previous findings (Ali 2008). Kamel et al. (2016) discovered that guava waste improved hot carcass weight compared to the control diet. Additionally, Morsy et al. (2019) found that the addition of guava leaf extract (1 or 3 ml/kg diet) improved the carcass percentage of growing rabbits. Adding 2% guava leaf meal to broiler feed can result in healthier, and low-fat meat (Daing et al. 2020, 2021).

Assessing the health and physiological condition of rabbits can be done by evaluating blood hematology. When adding a new feed additive, it is critical to assess changes in serum metabolites and blood profile to confirm the safety of the feed additive. The present study indicates that GLE-fed rabbits explained a significant increase in some blood indices (PCV and MCV) and a reduction in WBCs in diets containing 20 mg/kg GLE. As similar to the data reported in this research, Morsy et al. (2019) found that GLE reduced lipid levels (triglycerides, LDL, and total cholesterol) in growing rabbits after 8 weeks of feeding. There was no significant impact on blood hematology or total protein levels in rabbits, which aligns with our results. This finding suggests that the incorporation of GLE into the diet could improve the blood profile and prevent infections. In Indonesia, guava leaves are commonly used by locals to boost blood platelets in dengue fever patients and treat respiratory and gastrointestinal issues (Safitri et al. 2023). The mechanism behind this effect was not clear in this study and needs further clarification.

Serum metabolites are small molecules present in the blood that offer insights into health and metabolism. They can show the effects of stress, diet, and medications on metabolism. In this study, treated groups had normal levels of total protein, glucose, kidney markers, and bilirubin compared to controls. Adding GLE, particularly at 20 mg/kg diet, reduced total cholesterol, LDL, triglycerides, HDL, and VLDL in rabbit serum. The same results have been recorded in broiler-fed diets with 1-2% guava leaf meal (Daing et al. 2021). Quercetin found in GLE effectively regulates lipid profiles in rats with liver disease (Vijayakumar et al. 2018). The anti-lipogenic effect of GLE is attributed to gallic acid, which reduces lipogenesis by supporting cytosolic acetyl-CoA carboxylase 1. Guava leaves (GLs) are commonly used in traditional medicine for managing hyperlipidemia and hepatotoxicity in rabbits (Olaniyan et al. 2018). This function has been confirmed in this research, where a significant reduction in lipid profile was detected in rabbits fed diets with GLE. Moreover, flavonoids from GL extract have been linked to improved pancreatic beta-cell function and liver health in diabetic mice (Dewi et al. 2013). Flavonoids also inhibit the enzyme dipeptidyl-peptidase IV and prevent lipid accumulation and glucose uptake in cells without toxicity (Golovinskaia and Wang 2023). The antioxidant action of GLE is attributed to its diverse phenolic components, such as gallic acid, apigenin, ellagic acid, kaempferol, taxifolin, pyrocatechol, and ferulic acid (Kumar et al. 2021). Additionally, all of these compounds have shown various pharmacological activities including antioxidant, antimicrobial and immunomodulatory effects (Kumar et al. 2021). A previous research has found that the levels of TAC in the blood were improved, while the level of MDA was significantly decreased by the addition of GLE to growing rabbit diets (Morsy et al. 2019). This suggests that GLE has strong antioxidant activity. The antioxidant properties of guava extract were further evidenced by its ability to reduce lipid peroxidation, boost the antioxidant defense system, and decrease the generation of reactive oxygen species. Recently, a study by Hassan (2023) explored the role of GLE as a natural antioxidant. The study involved administering aqueous GLE (1g/dl) to streptozotocin (STZ)-induced diabetic rats for one month. The results showed an improvement in the activity of endogenous antioxidant enzymes such as GPx, SOD, and TAC. They attributed the potential hypoglycemic and antioxidant activities of GLE to the attendance of a relatively high percentage of phenolic compounds (456±10.4 mg gallic acid equivalent/l) and other active volatile molecules with remarkable antioxidant action. Daing et al. (2021) informed that incorporation of guava leaves (2-4%) meal substantially increased CAT, glutathione, GPx and SOD activities, and decreased MDA in broiler. Gallic acid reduces the excessive production of mitochondrial ROS in the liver by enhancing mitochondrial function (Jin et al. 2018). The present study also identified catechins in GLE. Catechins are known to effectively scavenge OS and free radicals by binding to lipids, proteins, nucleic acids, and metals in tissues. The biological activities of catechins are primarily due to the presence of at least 5 hydroxyl groups in the diphenylpropanoid skeleton (C6C3C6) structure. These structural features influence the antioxidant capacity of catechins (Kim et al. 2022). Feed additive supplements in the livestock industry have been used to enhance the immune response in rabbits (Saghir et al. 2023). A note of this, assessing the immune response reflects the animal’s ability to defend themselves. So, it’s critical to assess the immunoglobulins profile (IgA and IgM) in the serum of growing rabbits after feeding GLE. Based on the chemical constitution of GLE, it’s a rich source of vitamin C, which supports a healthy immune system and has also antimicrobial properties that help fight off infections. In broiler trial, Daing et al. (2021) shown that tannins isolated from guava leaves enhanced the immune system. Supplementing growing diets with medicinal plant extracts has been supported to enhance their growth, immunity, appetite, stress tolerance, and disease resistance in animals (Abubakar et al. 2023).

Improving intestinal health during the post-weaning period could enhance the survival of kids by reducing diarrhea and improving nutrient digestibility. In this study, the intestines of GLE-treated groups showed an enhancement in villous height, a moderate number of villi with branching, and more elongated and branched villous epithelium compared to rabbits fed a control diet. Wang et al. (2021a) found that dietary supplementation with 50–200 mg kg^−1^ GLE lightened the damage of intestinal mucosa induced by *E. coli* in piglets compared with the control group. Gallic acid in GLE can enhance the absorption of specific cationic amino acids by intestinal cells by regulating *CAT-1* expression (Tretola et al. 2021). This indicates that gallic acid improves protein and amino acid absorption across the intestinal barrier. Guava leaves extract has demonstrated beneficial effects on intestinal health by improving intestinal permeability, reducing mucosal damage, and promoting the integrity of tight junctions (Wang et al. 2021a; Wang et al. 2021b). This offers a promising approach for safeguarding and preserving the function of the intestinal barrier in growing rabbits. The liver plays a critical role in various physiological functions. GLE can improve liver function by reducing liver enzymes through its hepatoprotective effect (Olaniyan et al. 2018). The improved liver tissue integrity may be attributed to GLE's ability to reduce lipid accumulation in hepatocytes, thereby decreasing lipid oxidation. Some bioactive compounds found in GLE such as gallic acid can activate the AMPK signaling. AMPK is a key cellular energy sensor with multiple functions and stimulating the catabolic pathways (such as fatty acid oxidation and glycolytic pathways), thus reducing lipid accumulation in liver tissues (Zhang et al. 2023).

## Conclusion

The diets supplemented with GLE (20 mg/kg diet) improved the growth performance, feed utilization, blood physiology, and overall health of growing rabbits. Furthermore, dietary supplementation with GLE extract significantly reduced blood lipid levels and oxidative stress, while enhancing immune-ability and antioxidant activity in growing rabbits. These positive effects were also observed in the intestinal and hepatic architectures of rabbits fed diets containing GLE. This study suggests that utilizing agro-industrial waste like GLE in rabbit farms could offer a sustainable and environmentally friendly strategy.

## Contributions

Islam G. Abdalgahni and Asmaa M. Sheiha: Conceptualization, software, data curation, investigation. Mohamed F. Abo El-Maati and Islam G. Abdalgahni: Conceptualization, supervision, software, formal analysis, validation, visualization, methodology. Sameh A. Abdelnour: Validation, visualization, writing - original draft. Abdelhalim A. El-Darawany and Khaled M. Al-Marakby: Conceptualization, project administration, resources, supervision, software, formal analysis, Sameh A. Abdelnour and Khaled M. Al-Marakby visualization, writing - original draft, writing - review & editing.

## Data Availability

All data are available within the manuscript.
